# Addresses to Associated Bodies

**Published:** 1883-05

**Authors:** P. P. Wells

**Affiliations:** Brooklyn


### HOMŒOPATHIC PHYSICIAN,

A MONTHLY JOURNAL OF MEDICAL SCIENCE.

If our school ever gives up the strict inductive method of Hahnemann, we are lost, and deserve only to be mentioned as a caricature, in the history of medicine."—CONSTANTINE HERING.

Vol. III. MAY, 1883. No. 5.

### ADDRESSES TO ASSOCIATED BODIES.

P. P. WELLS, M. D., BROOKLYN.

It has been the endeavor of men holding similar views in matters of science, religion, or politics to give additional force and currency to those views by the power of association. They have gathered in bodies, that by this power they might aid each other in attaining increased knowledge of the principles they advocate, and gain, as in practical medicine, increased facilities in their administration in the practical duties of healing. Such associations may have power for good when, as bodies, they confine their endeavors to the legitimate objectives of their being, and not otherwise. Outside of these their utterances have only the force of individual opinion. The same is true of the utterances of their executive officers. When these leave the objectives of the association, they cease to carry with them more than the individual character of the speaker or writer, and cannot wield the authority of that body, the official head of which the speaker may chance to be at the time. His *opinions* are his own and not necessarily those of the association at all. This should be borne in mind when we read addresses of presidents of institutes or of societies. They are of no authority as ennunciations of the embodied opinions of those to whom they may have been speaking. They are often found to be far from the philosophy accepted and practiced by the body addressed.*

* At the meeting of the American Institute in St. Louis in 1868, several of the members expressed the opinion that they had listened to addresses

In a late number of the New York *Tribune* is found what purports to be an address by the president of the New York State Homœopathic Medical Society, delivered to its members assembled at Albany in its annual meeting, February, 1883. It is our purpose now to look into some of the utterances of this address and see, if we may, how far they will bear examination in the light of homœopathic law, experience, and duty. It will be remembered this society is supposed to be a homœopathic society—*i. e.*, a society founded on homœopathic law, and its members are, by the fact of their membership, supposed to profess allegiance to that law, and its president, by his official position, to be giving impulse to confidence in this accepted law, and helping by all his powers to its more perfect understanding and to a more complete comprehension of its requirements when applied for the cure of the sick. These are supposed to be chief objectives of the society and its president in their associated organization. We shall endeavor to see how far some of these utterances of the president tend to attaining these objectives. He says:

"The dead past we have buried. The living present, with all its truths increased intelligence, methods, and appliances, is ours. In becoming homœopathists we have never yielded our rights to use any or all of these * * * as our patients shall * * * require them for their comfort or cure."

The trouble with the writer of this paragraph, it would seem, is that he has not yet become a homœopath, and judging from this and some other paragraphs of this address, he has no very clear notion of what is involved in becoming one. The homœopath is supposed to have accepted God's law of healing as the basis of the only known science of therapeutics. Being God-given and God-enacted, the homœopathist may be supposed to have found it, in practical duties and results, so far superior to all mere human imaginings of methods or means of cure ("increased intelligence, methods,

---

enough on such occasions as that at the meeting at which they were then present, and the result was Professor Ludlam, of Chicago, moved, and the writer of this seconded, a resolution to dispense with addresses from presidents of the Institute at future meetings, or to restrict their expressions to discussions of a scientific character—*i. e.*, that hereafter such addresses should be brought strictly into accord with the objectives of the Institute's existence. Has this resolution been repealed? The originators of it thought they had listened to enough of such as the members of that body had then hitherto been accustomed. Does the Institute need another constitution?

and appliances") as to have no need of help from these, and that attempts to supplement the requirements of the law by any of these can only be injurious to results possible under a strict administration of our law, if not even destructive of both these and the patient. These attempts, the homœopathists know, can never serve either the "comfort or cure of their patients" when intruded into a genuine homœopathic practice.

Then, as to having "buried the dead past"—this is all a mistake. "The past," the president may be assured, is not "dead," and neither he nor his fellow-members can bury it. It lives in the present, or ought to live and be the teacher of societies and presidents, showing them in the practical history of Hahnemann, Bœnninghausen, Stapf, Gross, Haynel, and a host of others, that all twaddle about the right to use "means and methods" outside of those required by an intelligent administration of our law are only hurtful impediments to best success in healing, and are never called for to secure the best "comfort" and safety of patients. It is only too evident this "past" has never been studied or has been all too early forgotten, or we should have less of this prating of "liberty" and "palliation" outside of law, in our society addresses and discussions. The "past" is not "dead," only forgotten where it should be remembered. It is not dead, but living and eloquent in its instructions and rebukes of all such inculculations of practical heresies. It should never be forgotten that our school of practical medicine is a school founded on law. Law knows no "liberty" of violating its enactments. Law knows no liberty but the liberty of obedience. "Liberty of opinion!" Who has the liberty of an opinion which violates divine law? And who in all the universe could be benefited by such an opinion, if such were possible? Those who claim the right to such an opinion have never come under the dominion of law. The right they claim is only the right of transgressors, which is only the right of punishment. These have never "become homœopathists."

"In regard to the resolution offered by the 'regulars' last year, * * * we hailed it as a good omen. * * * We knew that we could do them good in the department of the practice of medicine to which for half a century we had especially devoted ourselves."

It would be interesting to know just how we were to do them good, we being the subjects of law and they repudiating all law. How we could do them good, except it should be by going outside of law, is certainly not very clear. And how this treason to law on our part is to help them is not more obvious. There is no way perceptible in which a disciple of law can help the repudiator of law, except he abandons his antagonism of law and comes voluntarily under its light, teachings, and authority. We have seen no evidence that these old-school "regulars" were in any haste to do this. The expectation of being able to help these worthies on any other foundation would seem to be baseless, and on this to be possible only with the extra hopeful.

"And then they could be of service to us in the various departments of surgery, pathology, hygiene, and climatology."

How help us? Are we ignorant of these knowledges that we require to be taught by our enemies, who affect to hold us in so great contempt? If we be so ignorant, then there is no doubt we have abundant cause for shame. The defect should be cured. But there is certainly a pleasanter way out of our disgrace than a resort to these long-time revilers of our philosophy and slanderers of our professional character. Was any suggestion ever more humiliating? If any of our school feel in need of this help we have no hesitation in saying he should either get out of his disgrace or out of our school.

"It is an entire fallacy to assert that a consultation cannot be held with a physician holding diverse views from your own without a compromise of your position or a sacrifice of your honor. That is, if the consultation be intended to advance the interests of the patient."

This will depend very much on the nature of the "*diverse views.*" If they chance to be the exact opposites of each other, as in the case of philosophy and practice founded on law on the one side, and on the other on utter rejection and disregard of all law, the very idea of a consultation between the two, "to advance the interests of the patient," would seem to be stupid and absurd to the last degree, there being in the therapeutics of the two supposed consultants no common ground in all the domain of common sense on which both can stand. This is not a question of "compromise of position" or "sacrifice of honor," but of a sacrifice of self-respect and common sense in the proposed "compromise" precedent to the supposed consultation. This is how an old-school physician talks and illustrates this matter of consultations between members of his school and ours,* and we do not see why his talk is not quite reasonable.

---

* Daniel Brown, M. D., New York city.

"If the homœopathists have principles of practice they should be laid down in their authoritative works, such as the *Organon*. Let us suppose a patient to have peritonitis. His homœopathic doctor is willing to consult. His wife, we will suppose, is sufficiently frightened to lay aside her love for those dear little bottles and try one of those horrid old-school men. Now, suppose I consent to go. There is no question of diagnosis; that point is settled before I get there. What the patient wants is the best remedies to relieve the excruciating pain and to cure. The 'aids of physiology and pathology,' as well as experience, teach me a plan of treatment that is directly contra-indicated by homœopathic books and principles. I do not believe the patient can get well without opium.* The other doctor believes that he can. We have a quarrel. I want my fee, and the patient gentlemanly pays it; but for what? Did he get value received for his money?"

How could the farce result otherwise, the two worthies having no common ground on which to stand. The doctor may be assured the "homœopathists" have principles of practice laid down in their "authoritative works," and plenty of them, and these are just what is the matter which gives the character of a farce to these much talked of consultations. And just here the question may not be impertinent —would it not have been as well for our old-school enemies, before talking so much for and against consulting with homœopathists, to have informed themselves whether homœopathists would consent to a consultation with *them*.† It is barely possible they might thus have discovered the repugnance to entering on this duty is not confined to their own side. It certainly is possible that there may be those in the homœopathic ranks who have as clear a perception of

* Dr. B. may be assured by one who has had more than forty years of practical experience and proof of the fact that peritonitis can be cured without opium, and better, much better, without than with. And more than this, after a comparison of the results of this forty years' dealing with this disease, with homœopathic remedies only, with his seven years with allopathic means only, he has no hesitation in saying peritonitis is more *certainly*, *speedily*, and *perfectly* cured by the *rightly selected* and *rightly administered* homœopathic remedy than by opium, or than by any or all other allopathic means together. We make this statement in view of a not very limited experience of the fact, both in our own person and in that of many patients. We know this the more certainly, having passed through the treatment of both methods.

† One is strongly reminded by this so largely one-sided talk of the story told by Fred Douglass of a very earnest but not over-refined would-be friend, who, after one of Mr. Douglass' brilliant speeches, came to him on the platform, and shaking him heartily by the hand, said: "Come, Fred, let us walk down Broadway arm in arm. I am not ashamed!" "He never thought of inquiring whether *I* should be ashamed," said this eminent orator.

and aversion to the farcical character of this proceeding as themselves. There may be found there such as would not feel themselves greatly honored by this kind of intercourse with their traditional and habitual enemies, and this all the more, as they know, if they need help (and who at times does not?), that there is no lack of *friends* from whom it may be had, who in the matter of intelligence are, to say the least, fully the equals of these, their loud-talking *enemies*. And further says Dr. Brown:

"But Piffard comes to the rescue here, and states they have abandoned those principles of treatment; that they treat diseases just as we do now. * * * If they have abandoned their principles and are simply trading on a name and deceiving the public, it is only a question of honor, and we desire only to protest by our silence."

If there be those in our ranks who have thus "abandoned their principles" and are thus playing the part of deceivers, they are unreservedly abandoned to all the condemnation and contempt Doctors B. and P. can heap on them, and if this be much these impostors deserve it all.*

And further from the address:

"Even in the treatment of diseases men of diverse views may approach a case and from their different standpoints come to a harmonious mode of procedure. The difficulty of doing this is more apparent than real. In the first place, we use all the drugs known to the old-school physicians."

We have sufficiently replied to this asserted facility of "*approach*" of those of "*diverse views*," and, unless we mistake, have shown the absurdity of the claim of the possibility. This absurdity is plain on the face of it, where, as in the supposed case of consultation, the "views" are direct opposites of each other. How can opposites approach each other? And the argument for these consultations is not helped in the least by the fact that "we use all the drugs known to the old-school physician." The difficulty is not so

---

*Another old-school doctor (Austin Flint, of New York) is reported to have advocated consultations with homœopathists, and to have given as his reason for his approval of them his desire to "come close" to these new-school men, that he "might stamp them out." No one is likely to doubt the sincerity or earnestness of the desire or the frankness of its expression. But while admiring these, one cannot but laugh at his imbecility as to his object in his approval. His *foot* is not large enough to "stamp out" either God's law of healing or its loyal adherents. In view of this desire, so frankly expressed, how about the "good omen"? Who will come first to be "stamped out"?

much in the possession or want of drugs as in the principle of their selection and use. And then, though we have and use all old-school drugs, we have and use beneficially many which the old school has not, and of which physicians of that school are utterly ignorant. Now, suppose in a consultation, on homœopathic principles, the case in hand requires for it some one of these unknown agents, how is the ignorance of one of the consultants to aid its discovery, or, if selected by the other, to help its efficiency? Or, in this case, is the homœopathist, who knows, to give up the specific remedy required for the cure in deference to the ignorance of his co-consultant who does not know? Let the advocates of these consultations answer.

And further from the address:

"Our doses can vary from the largest old-school potions to the highest of our infinitesimals without infringing at all upon our law of therapeutics."

Can they? Then what becomes of the corollary of our fundamental law, in the language of Hahnemann, "the least possible dose," more recently formulated, "*the minimum dose of the dynamized drug?*" Is there not in this utterance of our president strong confirmation of our early saying, he has not yet become a homœopath? *Most* certainly not much more than is contained in the name, and this is not much. And then:

"The constantly increasing use of our drugs in the cure of disease by the 'regular' physicians makes the matter still more easy of accomplishment, *i. e.*, consultation. Hundreds of our drugs are placed in their true relation to disease in allopathic works, through the use they have made of our provings and our works on the practice of medicine. And this process of assimilation of our discoveries is constantly going on."

We do not see how "the increasing use of our drugs" makes consultation easier, when the difficulty is not in the use of the drugs, but in the utter want of a knowledge of the principles of their right use on the part of one of the consultants. Nor is it plainer how these men can have "placed hundreds of our drugs in their true relation to disease in allopathic works," when this "true placing" is directed and governed by a law these allopathists reject. Does our president mean to insinuate that our enemies are "*trading*" on stolen wares, and to give this and the false pretense which goes along with this statement as a reason for consultation with them? Will he regard himself or his friends or his fellow-members of the State Society as honored by consultations with thieves?

Our president inquires:

"But can our 'regular' friend be of any use to us in treatment of cases? Well, yes, I presume he may. He understands the methods and appliances of palliative treatment better than we do."*

It must be a queer kind of help a practitioner who moves under the guidance of a divine law, receives from one who rejects this law, and from means which in their nature are in direct antagonism to this law. Is this a further evidence that our president has not yet become very *much* of a homœopathist?

And again:

"There are, of course, cases where our law fails or is inapplicable."

Why "of course," and what kind of cases are these, where God's law "*fails*" or "*is inapplicable*"? Did the maker of this law know what He was about when He ordained it? Did He or did He not know diseases, their nature, circumstances, and conditions, that He should, for want of knowledge or power, leave some outside the domain of His law? Our president may be assured the "failure" is in the administrator of the law and never in the law itself. "Failures" and "inapplicability" are wholly in those practitioners who have not yet become very much of homœopathists; indeed, not so much as they should before attempting the duties of the practical administration of the philosophy and law they profess to believe in and practice. We are, of course, speaking of curable cases of sickness. But for the incurable, even, we have no doubt the greatest possible relief for them is found in the remedy which is *most like.*

Our president says further:

"In such cases we may have to resort to old-school methods, for one cause or another. * * * Our law, far-reaching as it is, has its limitations."

In a homœopathic experience of more than forty years we have never found "*such cases*"—no, not even one, and do not in the least believe in them. In these forty years and more we have never in one instance resorted to "old-school methods" or means, have never seen any need of them, or any case of sickness where there was the faintest reason to believe these could in any way help the most similar remedy, or in any degree show themselves superior to this as

---

* For a discussion of this matter of palliatives and their relation to homœopathic practice, *vide* Hahnemann's *Organon of Homœopathic Medicine,* and THE HOMŒOPATHIC PHYSICIAN, Vol. III, p. 78.

curing agents. The "*limitations*" are in the *man* who attempts the performance of duties to which he is inadequate, and not in the *law* at all.

In dismissing these extracts from our president's address, we may remark, if they had been met as coming from a private, unofficial source, they would certainly have been sufficiently mortifying, but might have been permitted to pass without comment; but coming from a president of a society so numerous and conspicuous as the State Society of New York, they become a source of humiliation to intelligent homœopathists so great as to justify the most severe condemnation. Indeed, the reading of them could hardly fail of suggesting the question whether we have not suffered enough already from efforts in presidents' addresses to misrepresent, disparage, and belittle and subvert the authority and importance of our fundamental law and its philosophy to allow such addresses to stop? Have we not had enough, and more, of utterances such as we have felt our duty here to comment on to satisfy the cravings of our worst enemies? Our friends—have they not been made to suffer enough already to constitute a valid claim that they be spared in the future the shame and humiliation the past has so liberally heaped on them, and to justify them if now they ask that "*addresses*" may be permitted to stop?

