# Errata

**Published:** 1883-06

**Authors:** 


Errata: In our May issue, p. 142, last line, for *constitution* read *resolution.*

